# Immunogenicity at delivery after Tdap vaccination in successive pregnancies

**DOI:** 10.3389/fimmu.2024.1360201

**Published:** 2024-02-23

**Authors:** Louise De Weerdt, Anaïs Thiriard, Elke Leuridan, Arnaud Marchant, Kirsten Maertens

**Affiliations:** ^1^ Centre for the Evaluation of Vaccination, Vaccine and Infectious Diseases Institute, University of Antwerp, Antwerp, Belgium; ^2^ European Plotkin Institute for Vaccinology, Université libre de Bruxelles, Brussels, Belgium

**Keywords:** Tdap, successive pregnancies, maternal antibodies, IgG subclasses, transplacental transfer

## Abstract

**Background:**

Tetanus, diphtheria, acellular pertussis (Tdap) vaccination is recommended to be administered in every pregnancy. Although the safety of this strategy has been confirmed, the immunogenicity of Tdap vaccination in two successive pregnancies has not yet been described. This study investigated Tdap-specific immunity levels and transplacental transfer in two successive pregnancies after repeated Tdap-vaccination.

**Methods:**

Women enrolled in prior studies on Tdap vaccination during pregnancy were invited to participate in a follow-up study if they became pregnant again. Women who received a Tdap vaccine in both pregnancies were considered for this analysis. Tdap-specific total IgG and IgG subclasses were measured with a multiplex immunoassay.

**Results:**

In total, 27 participants with a mean interval between deliveries of 2.4 years were included in the analysis. In maternal serum, Tdap-specific total IgG levels were comparable at both deliveries whereas in cord serum, all Tdap-specific total IgG antibody levels were reduced at the second compared to the first delivery. This was largely reflected in the IgG1 levels in maternal and cord serum. Transplacental transfer ratios of total IgG and IgG1 were also mostly reduced in the second compared to the first pregnancy.

**Conclusion:**

This study reports for the first time Tdap-specific total IgG and IgG subclass levels and transfer ratios after repeated Tdap vaccination in successive pregnancies. We found reduced transfer of most Tdap-specific IgG and IgG1 antibodies in the successive pregnancy. As pertussis-specific antibodies wane quickly, Tdap vaccination in each pregnancy remains beneficial. However, more research is needed to understand the impact of closely spaced booster doses during pregnancy on early infant protection against pertussis.

## Introduction

Despite the availability of universal pertussis immunization programs achieving high coverage, pertussis has re-emerged as an important respiratory infection during the last decade. According to Yeung et al., an estimated 24.1 million pertussis cases and 160,700 pertussis-related deaths in children younger than 5 years old occur annually (1). The most affected population are infants (2), too young to be completely protected by the currently available vaccines and vaccination schedules (3).

A three-dose primary series of diphtheria-tetanus-pertussis vaccines is recommended to be given from 6 weeks of age onwards (4). As immunity against pertussis after vaccination or natural infection wanes over time, repeated pertussis booster doses are needed throughout life to prevent infection and to protect vulnerable populations such as unvaccinated infants by reducing transmission. A single booster dose of tetanus, diphtheria, acellular pertussis (Tdap) is therefore recommended by the Advisory Committee on Immunization Practices for persons aged 11 to 18 years. From the age of 19 onwards, a booster dose of either Td or Tdap is recommended to be administered every 10 years throughout life to ensure continued protection (5). The safety and immunogenicity of this decennial Tdap booster in adolescents and adults have been established in previous research (6–8). Tdap immunization less than 2 years after tetanus vaccination was also found to be safe in the general population (9, 10).

To ameliorate the protection of vulnerable infants in their first weeks of life, Tdap vaccination is recommended during pregnancy in an increasing number of countries (5, 11). In-pregnancy vaccination elevates the levels of disease-specific maternal antibodies in pregnant women which are then transferred to the newborn through transplacental transport and breastfeeding and provide passive protection to the newborn in the first weeks postpartum (12). Although a correlate of protection for pertussis is not yet defined, high levels of immunoglobulin G (IgG) antibodies against pertussis toxin (PT), pertactin (PRN), and filamentous hemagglutinin (FHA) are known to be important effectors to mediate protection (13). IgG antibodies can be further divided into four subclasses, IgG1, IgG2, IgG3 and IgG4, that structurally differ in their constant region resulting in different effector functions, half-life and transplacental transport (14). Typically, IgG1 and IgG3 are potent inducers of Fc-mediated effector mechanisms, whereas IgG2 and IgG4 have lower Fc-dependent effector potential. Immunoglobulin class switching involves the change of B cell’s antibody production from one isotype to another and allows the immune system to engage with each antigen in a specific manner with unique effector mechanisms being imprinted by each (sub)class (15). The transport of IgG subclasses across the placenta is known to be mediated by the neonatal Fc receptor (FcRn) with preferential transfer of IgG1 and less efficient transfer of IgG2, IgG3 and IgG4, although there is no absolute consensus in the hierarchy of subclass transfer efficiency (16).

Vaccine-induced pertussis-specific antibodies are known to wane quickly (17–19). Therefore, vaccination is recommended during each pregnancy, maximizing the maternal antibody response and passive antibody transfer to adequately protect the newborn regardless of the interval between pregnancies (5). For pregnant women who receive Tdap during multiple closely spaced pregnancies, a theoretical risk for severe local reactions exists based on historical data on multiple doses of tetanus-containing vaccines (20). On the other hand, the potential benefit of preventing pertussis morbidity and mortality in infants should outweigh the theoretical concerns of possible severe adverse events. Moreover, more recent data did not find increased risks for acute adverse events and adverse birth outcomes after Tdap vaccination in pregnancy with a recent prior tetanus-containing vaccination (21, 22). The safety of Tdap administration in successive pregnancies within a 5-year timespan has also been confirmed (23). However, currently, the immunogenicity in successive pregnancies after repeated Tdap vaccination has not yet been described. In other words, it is not known if pregnant women achieve the same level of immunity in successive pregnancies after Tdap vaccination in both pregnancies and whether maternal antibodies are equally transported across the placenta. As vaccination in pregnancy is becoming an increasingly important strategy to protect infants, it is of importance to improve our knowledge concerning transplacental transfer of immunity and the impact of IgG subclasses on this transport.

The primary aim of this study is therefore to investigate Tdap-specific IgG antibody levels and the transplacental transfer of IgG antibodies from mother to child at two successive deliveries after Tdap vaccination in both pregnancies. The study also aims to evaluate Tdap-specific IgG subclass levels as well as the transplacental transfer of these antibodies.

## Methods

### Study design

Women enrolled in prior studies on pertussis vaccination during pregnancy (clinicaltrials.gov NCT01698346 and NCT02511327) were invited to participate in a follow-up study when they became pregnant again. Women who received a licensed Tdap vaccine in both pregnancies (Boostrix^®^, GlaxoSmithKline or Triaxis^®^, Sanofi Pasteur; depending on availability within the national vaccination program at the moment of vaccination) were included in the follow-up study. Vaccination was planned and performed according to protocol by a study physician or within the regular healthcare system following the current Belgian recommendation, i.e. vaccination between 24 and 32 weeks of gestation, if feasible (24). A maternal and cord blood sample were collected from all mother-infant pairs at each delivery. Cord blood samples were collected immediately after birth; maternal blood samples were collected within 72 hours after delivery. All collected samples were centrifuged and serum was stored at -35°C until further use.

The pregnancy of the baseline studies was not for all participants their first pregnancy but will be further referred to as ‘pregnancy 1’ and ‘delivery 1’ in this paper. The pregnancy of the follow-up study succeeded the previous pregnancy and will further be referred to as ‘pregnancy 2’ and ‘delivery 2’.

For both baseline studies and the follow-up study, a questionnaire on demographics, vaccination history and general medical history was collected. Growth parameters of the newborn, breastfeeding data, daycare attendance, immunization data, and medical histories for all household members were collected at each visit. Full details on inclusion and exclusion criteria can be found in the publications of the original studies (25–27).

### Tdap-specific IgG and IgG subclass detection

All available leftover maternal and cord serum samples from both deliveries were tested at the Université Libre de Bruxelles (ULB). Total and subclass Tdap-specific IgG antibodies were quantified using an *in-house* bead-based multiplex immunoassay (28). In brief, serum samples were incubated with distinct fluorescent magnetic beads, individually coated with specific purified target antigens: PT (List Labs, #180), FHA (Sigma, #F5551), PRN (NAC, #NAT41666), diphtheria toxin (DT, List Labs, #151) and tetanus toxin (TT, MassBiologics, # LP1105P). Samples were measured at appropriate dilutions with a Multigam reference (CAF-DCF, Belgium), a WHO international standard NIBSC 06/140 and blanks included on each plate. Serum dilutions used to titrate IgG and IgG1 were 1:9000-1:18000-1:36000, 1:70-1:140-1:280 for IgG2 and IgG3, and 1:70-1:700-1:7000 for IgG4. After washing, the captured IgG was detected with R-phycoerythrin labelled antibodies specific for each isotype (IgG1-4).

Antigen-antibody binding was read on a BioPlex-200 (Bio-Rad, California, USA) obtaining mean fluorescence intensity (MFI). Tdap-specific total IgG antibody MFI levels were converted into international units per milliliter (IU/mL) by interpolation from a weighted five-parameter logistic standard. The lower limit of quantification (LLOQ) was defined as the lowest concentration within the linear part of the standard curve and corresponds to the mean of the interpolated bottom best-fit values of all plates, multiplied by 2. Adjusted LLOQ concentration for PT, FHA, PRN, DT and TT was 6.9, 26.0, 7.2, 0.04 and 1.9 IU/mL respectively. For Tdap-specific subclass levels, the MFI results of the different dilutions were combined to generate an area under the curve (AUC) measurement.

### Statistical analysis

No sample size calculation was performed since convenience sampling was done, reaching out to samples from participants of prior maternal pertussis vaccination studies with a successive pregnancy. Descriptive analyses were performed to identify possible demographic or clinical differences between both pregnancies. Statistical tests included paired *t* tests for continuous data and chi-square tests for categorical data.

Antibody levels were presented as geometric mean concentrations (GMC) with 95% confidence interval in IU/mL (Tdap-specific total IgG) or median AUC with interquartile ranges (Tdap-specific IgG1-4). Comparisons of antibody levels in maternal and cord serum between the successive pregnancies were performed with a paired Wilcoxon signed-rank test (non-parametric). The transplacental transport ratio (TTR) was calculated as the ratio of cord and maternal serum antibody levels. Calculated ratios were corrected with the ROUT method to identify outliers (Q=0.1%). Statistical significance between delivery 1 and delivery 2 was set at p <0.05 for each analysis (* p < 0.05, ** p < 0.01, **** p < 0.0001). A linear regression was applied to investigate the impact of time interval between Tdap vaccinations on the difference in TTR between deliveries. The analyses were performed using GraphPad Prism, version 10.1.0.

## Results

### Study population

**Figure 1:**
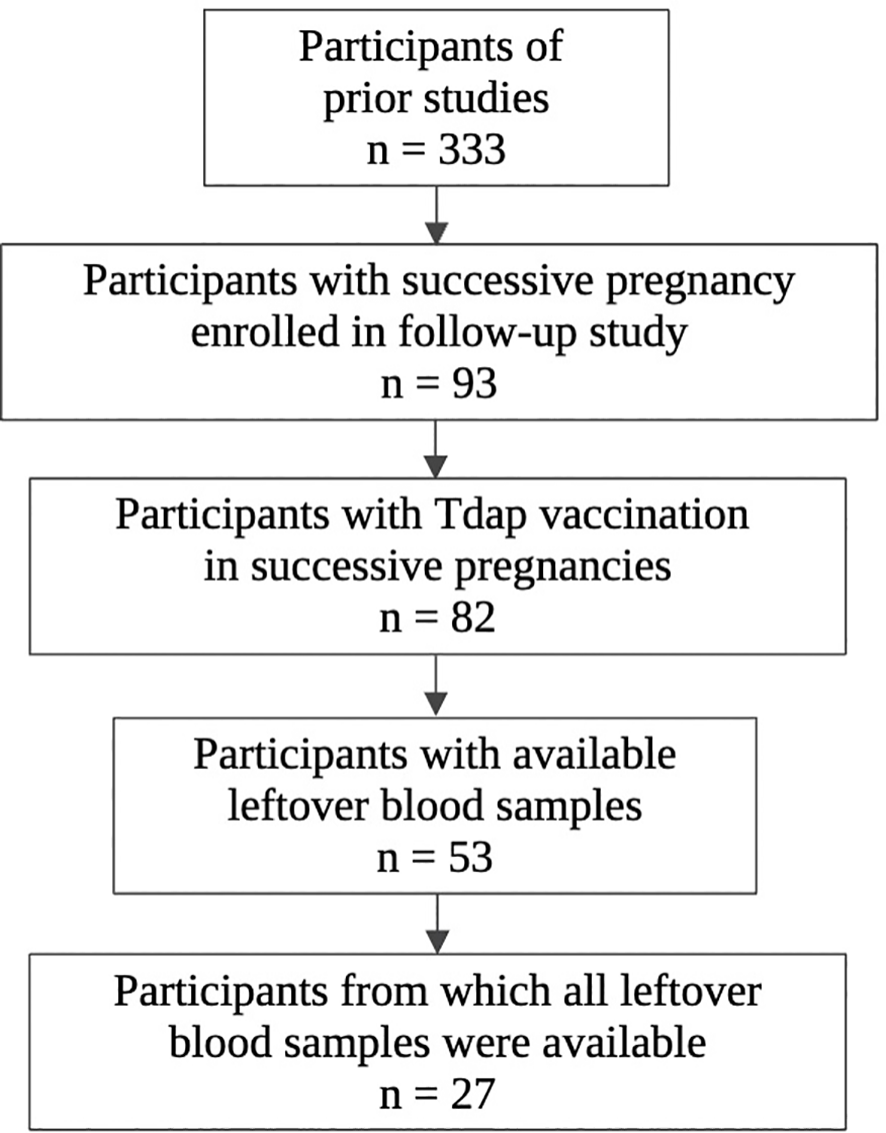
Flowchart of selection procedure participants. Created in Biorender.com.

The selection procedure of the participating women and the number of available serum samples are shown in **Figure 1**. A total of 333 participants were enrolled in the baseline studies mentioned earlier. Of these, 93 women enrolled in the follow-up study with a successive pregnancy. Tdap vaccination in both pregnancies could not be confirmed for 11 women, who were excluded from this analysis. Of 82 participants, leftover maternal and cord serum at both deliveries were available from 27 mother-infant pairs. The mean interval between delivery 1 and delivery 2 was 2.4 years (1.4-3.9). None of the participants had a record of pertussis disease within the past 5 years prior to participation. Other demographic characteristics are presented in **Table 1**. No significant demographical differences were present in pregnancy 1 versus pregnancy 2, except for parity and pertussis vaccine brand. These differences are expected since parity is linked to the succession of pregnancies and since the Triaxis vaccine was not yet offered by the Belgian health care system during recruitment of pregnancy 1.

**Table 1:** Demographic characteristics of participating women.

MOTHER	No (%)		
Participants, no.	27 (100.0)		
Race, no. (%) Caucasian Other	27 (100.0) 0 (0.0)		
Last Tdap vaccination before Tdap vaccination in pregnancy 1, no. (%) < 10 years ≥ 10 years Unknown	5 (18.5) 11 (40.7) 11(40.7)		
Last Td vaccination before Tdap vaccination in pregnancy 1, no. (%) < 10 years ≥ 10 years Unknown	12 (44.4) 9 (33.3) 6 (22.2)		
Interval between delivery 1 and 2, *years* (SD) < 2.5y ≥ 2.5y	17 (63.0) 10 (37.0)		
PREGNANCIES	Pregnancy 1	Pregnancy 2	*p* value
Mode of delivery no. (%) Vaginal C-section Unknown	22 (81.5) 5 (18.5) 0 (0.0)	17 (63.0) 5 (18.5) 5 (18.5)	0.06
Parity, no. (%) 0 1 2 3	20 (74.1) 6 (22.2) 1 (3.7) 0 (0.0)	0 (0.0) 20 (74.1) 6 (22.2) 1 (3.7)	<0.0001
Influenza vaccination during pregnancy, no. (%) Yes No Unknown	13 (48.1) 9 (33.3) 5 (18.5)	7 (25.9) 7 (25.9) 13 (48.1)	0.06
Pertussis vaccine brand, no. (%) Boostrix Triaxis Unknown	27 (100.0) 0 (0.0) 0 (0.0)	19 (70.4) 5 (18.5) 3 (11.1)	0.01
Twin pregnancies, no. (%) Yes No	6 (22.2) 21 (77.8)	2 (7.4) 25 (92.6)	0.13
Premature delivery, no. (%) Yes No	2 (7.4) 25 (92.6)	1 (3.7) 26 (96.3)	0.55
Mean gestational age at vaccination, *weeks* (SD)	29.5 (2.9)	28.8 (2.6)	0.34
Mean gestational age at delivery, *weeks* (SD)	39.6 (1.5)	39.5 (1.5)	0.64
Mean interval between vaccination and delivery, *days* (SD)	70.9 (24.5)	74.3 (19.9)	0.52
Mean interval between vaccination and delivery, *weeks* (SD)	10.1 (3.5)	10.6 (2.8)	0.51

### Laboratory results

#### Tdap-specific IgG levels at successive deliveries

**Table 2** summarizes the concentrations of IgG antibodies to PT, FHA, PRN, DT and TT in maternal peripartum and cord serum samples from the successive pregnancies. In maternal serum, IgG levels against all tested antigens were comparable at the first and second delivery. In cord serum, levels were significantly lower for all antigens tested at the second compared to the first delivery. At the first delivery, additional analysis also showed significantly higher IgG levels in cord compared to maternal serum, whereas at the second delivery, this was only true for anti-FHA and anti-PRN levels. These results suggest reduced efficiency of transplacental transport of Tdap-specific IgG antibodies after Tdap-vaccination in successive pregnancies. On the other hand, seroprotective levels for diphtheria and tetanus, defined as antibody concentrations ≥ 0.1 IU/mL, were reached at both deliveries for all participants. For pertussis, no correlate of protection currently exists. Active IgG transport from mother to fetus in pregnancy was observed in our study for all tested antibodies in the first pregnancy but was not present or less pronounced during the second pregnancy.

**Table 2:** Geometric mean concentrations with 95% confidence intervals for Tdap-specific total IgG in maternal and cord serum at successive deliveries expressed in IU/mL.

	GMC (95% CI)					
	Maternal			Cord		
	Delivery 1	Delivery 2	*P^a^*	Delivery 1	Delivery 2	*P* ^a^
**Anti-PT**	85.0 (66.2-109.2)	90.3 (71.1-114.7)	0.84	114.1 (89.7-145.0)	59.8 (33.7-106.1)	0.049
**Anti-FHA**	401.6 (313.6-514.2)	343.6 (276.7-426.7)	0.14	557.0 (428.9-723.4)	339.1 (232.2-495.1)	<0.001
**Anti-PRN**	695.3 (471.5-1025.0)	693.7 (497.1-968.1)	0.44	957.5 (661.1-1387.0)	946.6 (687.9-1303.0)	0.04
**Anti-DT**	2.3 (1.8-2.8)	2.2 (1.8-2.8)	0.75	3.1 (2.4-4.0)	2.2 (1.6-3.0)	0.02
**Anti-TT**	15.2 (12.6-18.3)	15.3 (13.0-18.1)	0.64	19.6 (16.7-23.2)	16.5 (14.3-19.1)	0.02

PT, pertussis toxin; FHA, filamentous hemagglutinin; PRN, pertactin; TT, tetanus toxoid; DT, diphtheria toxoid; ^a^By the paired Wilcoxon signed-rank test.

#### Transplacental transport of Tdap-specific IgG in successive pregnancies

To further explore transfer of Tdap-specific IgG in successive pregnancies, the TTR was calculated for both pregnancies. The geometric mean TTR of total IgG was reduced at the second compared to the first delivery for all antibody specificities tested. This reduction was significant for antibodies against DT and TT (**Table 3**).

**Table 3:** Geometric mean concentration (GMC) transplacental transport ratios (TTR) with 95% confidence intervals (CI) of Tdap-specific IgG antibodies successive deliveries.

	GMC (95% CI)		
	Delivery 1	Delivery 2	*P^a^*
**Anti-PT**	1.3 (1.2-1.6)	0.7 (0.4-1.1)	0.06
**Anti-FHA**	1.4 (1.2-1.6)	1.0 (0.7-1.4)	0.14
**Anti-PRN**	1.4 (1.2-1.7)	1.2 (1.0-1.5)	0.71
**Anti-DT**	1.4 (1.2-1.6)	1.0 (0.8-1.2)	0.03
**Anti-TT**	1.3 (1.1-1.5)	1.1 (0.9-1.2)	0.05

GMC, geometric mean concentration; 95% CI, 95% confidence interval; PT, pertussis toxin; FHA, filamentous hemagglutinin; PRN, pertactin; TT, tetanus toxoid; DT, diphtheria toxoid; ^a^By the paired Wilcoxon signed-rank test.

#### Impact of time interval between Tdap vaccination on transplacental transport difference

**Figure 2:**
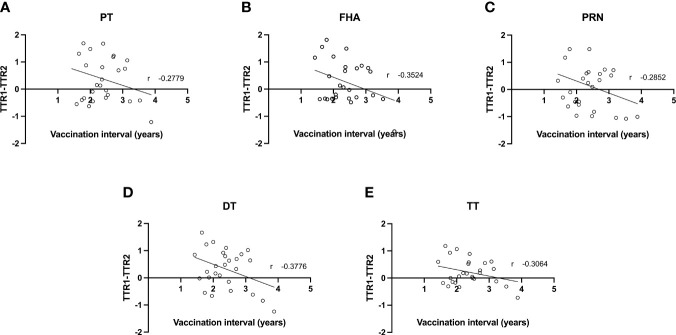
Correlation between difference in transplacental transport ratio of delivery 1 (TTR1) and delivery 2 (TTR2) and the interval between Tdap vaccinations in successive pregnancies of antibodies against PT **(A)**, FHA **(B)**, PRN **(C)**, DT **(D)** and TT **(E)**.

As the mean interval between Tdap vaccination in the successive pregnancies of the participants amounts 2.4 years (1.4-3.9) similarly to the interval between deliveries, all participants received a closely spaced Tdap booster in their successive pregnancy. The difference between the TTR of the first versus second pregnancy was further investigated with the time interval between Tdap vaccination in the successive pregnancies as a continuous variable (**Figure 2**). A weak negative correlation (between -0.2 and -0.39) between vaccination interval and difference in TTR was found for all Tdap-specific antibodies tested. This weak trend suggests a greater decrease in TTR between pregnancies with a shorter interval between vaccinations.

#### Levels of Tdap-specific IgG subclasses at successive deliveries

**Figure 3:**
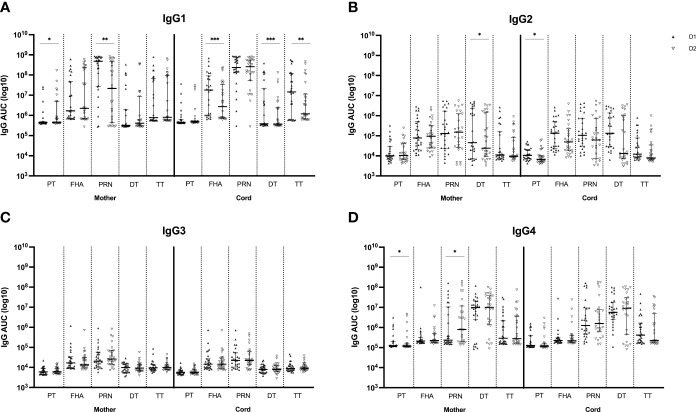
Tdap-specific serum levels of IgG1 **(A)**, IgG2 **(B)**, IgG3 **(C)** and IgG4 **(D)** in maternal and cord serum at successive deliveries. Results are expressed in log_10_ AUC and plotted as median with interquartile ranges. Statistical significance between delivery 1 (D1, dark labels) and delivery 2 (D2, white labels) was determined by a paired Wilcoxon signed-rank test and set a p<0.05 (*p<0.05, **p<0.01, ****p<0.0001).

The IgG subclass distribution was tested subsequently in the same serum samples for the same Tdap-specific antibodies (**Figure 3**). IgG1 subclass levels were comparable at first versus second delivery in maternal serum for anti-FHA, anti-DT and anti-TT antibodies but were significantly lower at the second delivery for anti-PT and anti-PRN antibodies. In cord serum, IgG1 subclass levels were significantly lower at the second compared to the first delivery for anti-FHA, anti-DT and anti-TT. The levels of IgG2 and IgG3 against all tested antigens remained stable in both maternal and cord serum across pregnancies except for significantly lower anti-DT IgG2 antibodies in maternal serum and anti-PT IgG2 in cord serum at the second delivery. IgG4 levels in maternal serum were significantly lower for anti-PT at the second delivery, but significantly higher for anti-PRN. In cord, IgG4 subclass levels were comparable between deliveries.

#### Transplacental transport of Tdap-specific IgG subclasses in successive pregnancies

**Figure 4:**
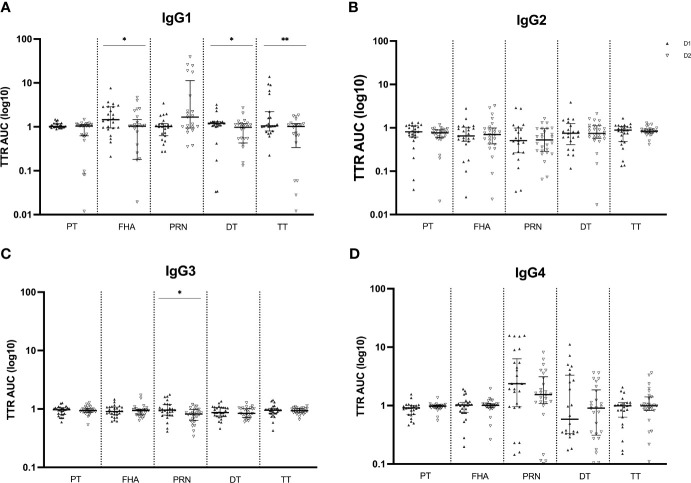
Transplacental transport ratio (TTR) of Tdap-specific IgG1 **(A)**, IgG2 **(B)**, IgG3 **(C)** and IgG4 **(D)** in maternal and cord serum at successive deliveries. Ratios are calculated using the log_10_ AUC results and plotted as median with interquartile ranges. Statistical significance between delivery 1 (D1, dark labels) and delivery 2 (D2, white labels) was determined by a paired Wilcoxon signed-rank test and set at p <0.05 (*p < 0.05, **p < 0.01).

Like the Tdap-specific total IgG levels, subsequent analysis of the IgG subclass distribution was conducted, calculating the transfer ratio of IgG1, IgG2, IgG3 and IgG4 across the placenta in successive pregnancies (**Figure 4**). Transfer efficiency was significantly reduced for IgG1 antibodies against FHA, DT and TT at the second compared to the first delivery. The transfer efficiency of IgG2, IgG3 and IgG4 antibodies did not differ among pregnancies, except for lower transfer efficiency of IgG3 anti-PRN antibodies in the second compared to the first delivery. On the other hand, although not significant, anti-PRN IgG1 transfer efficiently was higher in the second compared to the first pregnancy.

## Discussion

Even though Tdap vaccination is in most countries recommended during each pregnancy, immunogenicity data concerning Tdap vaccination in successive pregnancies are scarce. The present research assessed the impact of Tdap vaccination in two successive pregnancies on Tdap-specific total and subclass IgG levels in maternal and cord serum at delivery and the transplacental transport across the placenta.

In maternal serum at delivery, we found comparable Tdap-specific IgG levels at the first and second delivery whereas in cord serum, levels were lower at the second as compared to the first delivery. The lower cord serum levels were related to lower transfer ratios of these antibodies at the second delivery. To our knowledge, this is the first paper to report immunogenicity data at successive deliveries after Tdap vaccination in both pregnancies. The immunogenicity of the repeated decennial Tdap booster doses has been studied before in the general population and showed similar Tdap-specific antibody responses after first versus the second booster dose (6, 7). These results are in line with the antibody levels in maternal serum in our study that reach similar levels at both deliveries even though the mean interval between both deliveries amounts 2.4 years in contrast to a 10-year interval for the general booster doses. Another study in the US investigated the immunogenicity of Tdap vaccination in pregnancy with self-reported prior Tdap receipt (29). They reported significantly higher baseline Tdap-titers in pregnant women with prior Tdap and significantly lower post-vaccination titers against FIM, PRN and TT. Half of these women had received prior Tdap in the past 1-5 years. The study suggested that recent prior Tdap vaccination modifies the immune response after Tdap vaccination in pregnancy. However, the study did not measure transplacental transport of antibodies and their levels in newborns. In addition, prior Tdap could not be confirmed for a substantial part of the participants. The effects of prior influenza vaccination on maternal and cord serum antibody levels in pregnant women have also been studied before, with no significant differences reported at delivery (30). However, vaccination history in that study was again based on self-report and only vaccination in the prior year was determined.

The effect of a pre-pregnancy Tdap vaccination on antibody titers at delivery was studied before in which women received a Tdap booster in-between successive pregnancies instead of vaccination in pregnancy. Despite the significant increase in antibody titers between first delivery and one-month postvaccination, levels had declined significantly between postvaccination and second delivery, but were still significantly higher than baseline concentrations at first delivery. The mean interval between vaccination and second delivery was 16.8 months. Although baseline measurements were not included in our study design, these data suggest significant waning of antibody levels after a mean interval between Tdap vaccinations of 2.4 years in our study.

Unvaccinated pregnant women were not included in our analysis, making a direct vaccination versus non-vaccination comparison not possible. However, antibody levels at delivery from Tdap-vaccinated versus unvaccinated pregnant women have been reported before, with significantly lower Tdap-specific antibody levels in cord serum from both term and preterm born infants of unvaccinated compared to Tdap-vaccinated mothers (26, 27). The antibody levels in cord serum of both term and preterm born infants from these unvaccinated pregnant women are also considerably lower for each of the antigens tested compared to the antibody levels in cord serum in the successive pregnancy of our analysis. In addition, seroprotective total IgG levels for diphtheria and tetanus were measured at both deliveries for all participants. This indicates that infants of mothers vaccinated with Tdap in successive pregnancies would still be better protected against the disease compared to infants from unvaccinated mothers as seroprotective levels are more likely not to be reached in unvaccinated mothers. These observations underscore the importance of vaccination in each pregnancy.

Active IgG transport from mother to fetus during pregnancy, reaching higher concentrations in the newborn than in the mother, has been established before (31, 32) and was confirmed in our study in the first pregnancy. However, in the second pregnancy, the transfer efficiency was reduced. Antibody subclass is one of the factors that is known to modulate this efficiency as differences in transfer efficiencies have been noted across IgG subclass antibodies in prior research (14). In the present research, IgG subclass levels were measured to further evaluate possible differences at successive deliveries. Remarkably, IgG1 subclass levels in cord serum were lower at second compared to first delivery for most antibodies tested, in accordance with the total IgG data in cord serum. These reduced IgG1 levels suggest reduced Fc-mediated effector functions such as antibody-dependent cellular cytotoxicity (ADCC), complement-dependent cytotoxicity (CDC), and antibody-dependent cellular phagocytosis (ADCP). Since IgG1 is the most abundant IgG subclass in human sera, constituting more than 60% of the total IgG, it makes sense that the IgG1 results are in line with total IgG (33). Our data indeed also show superior IgG1 levels compared to the other three subclasses, with the lowest levels seen for IgG3. However, in maternal serum at delivery, IgG1 levels do not completely match the total IgG results as lower anti-PT and anti-PRN IgG1 levels were detected at second versus first delivery whereas total IgG levels were comparable between deliveries. In theory, the sum of the IgG subclass levels should be equal to the total IgG level for each of the antibodies tested. One could therefore reason that these differences outbalance each other, such as the decrease of IgG1 PRN antibodies versus the increase of IgG4 PRN antibodies in maternal serum at second versus first delivery. Such a shift in subclass may be related to more advanced class switch to IgG4 in PRN specific memory B cells following repeated antigen exposure, as previously described, although this shift was not seen for the other antigens (14). Such reasoning should be handled with caution though because different secondary monoclonal antibodies have been used for the distinct subclass assays and no quantification of IgG subclasses was performed. Finally, the reduced IgG1 levels in cord serum are also reflected in the impaired transfer efficiency of the majority of the IgG1 antibodies tested, possibly related to reduced affinity to the FcRn or to other receptors involved in transfer. Interestingly, despite the significantly lower level of total IgG and IgG2 anti-PT in cord serum and its lower total IgG TTR at the second delivery, the anti-PT TTR is not distinctly impaired at the second compared to the first delivery for any of the IgG subclasses.

Other factors besides antibody subclass may also modulate the transplacental antibody transfer efficiency, such as antigen specificity, Fc glycosylation and functional profile (34). A recent study reported similar antibody Fc-dependent effector responses in pregnant versus non-pregnant women following Tdap vaccination (35). They also observed similar memory B cell responses in both study groups and thereby inferred that the induction of vaccine responses in successive pregnancies is not affected by preceding pregnancies. The functional profile would thus not be modified in successive pregnancies, although biophysical characteristics such as antibody avidity were not explored in that research. Pregnancy itself is associated with a physiological increase in IgG Fc galactosylation and galactosylated IgG were shown to be preferentially transported across the placenta (34, 36, 37). Diminished binding of deglycosylated IgG1 to the FcRn receptor has been reported by a German study and suggests that Fc glycosylation influences IgG1 interaction with FcRn to a certain extent (38). Different Fc glycosylation profiles for Tdap-induced IgG1 antibodies in successive pregnancies could thus potentially contribute to the reduced IgG1 transplacental transfer at the successive pregnancy observed in our study.

During pregnancy itself, immunological adaptations are modulated by increased estrogen and progesterone levels that are important for innate immune surveillance and tolerogenic responses (39). Progesterone is also known to enhance immunoglobulin class switching with increased antibody production due to an increased production of B cells (39). Hormone concentrations in successive pregnancies have been studied before, with lower estrogen and progesterone levels detected throughout pregnancy in multiparous compared to primiparous women (40). Vaccine responses in successive pregnancies might also be affected by these hormonal changes. Further research concerning the impact of Tdap vaccination in successive pregnancies on hormonal changes is needed to better understand the immunological implications of these changes.

Our study has some limitations. Since left-over samples were used, available mother-infant samples from successive deliveries were limited. Within the selected cohort, some mothers received a different Tdap vaccine in their second pregnancy. However, this proportion was too low to analyze its impact. Also, no blood sample was taken before vaccination in the first pregnancy nor before vaccination in the second pregnancy. A baseline reference and the degree of waning before vaccination in the second pregnancy are therefore lacking.

Up to our knowledge, this paper is the first to report immunogenicity data at successive deliveries after Tdap vaccination in both pregnancies. Our findings suggest that the transfer efficiency of Tdap-specific IgG antibodies and more specifically IgG1 is reduced after repeated Tdap-vaccination in successive pregnancies. In other words, infants born at a close successive delivery seem to inherit fewer maternal antibodies compared to the infant born at the first delivery and might therefore be less protected. Seroprotective levels for diphtheria and tetanus at both deliveries for all participants indicate on the other hand that infants of mothers vaccinated with Tdap in successive pregnancies would still be better protected against the disease compared to infants from unvaccinated mothers. This highlights the importance of Tdap vaccination in each pregnancy. More research is needed to further explore the immunobiology of vaccine responses in pregnancy and the impact of closely spaced booster doses during pregnancy on early infant protection against pertussis.

## Data availability statement

The original contributions presented in the study are included in the article/supplementary material. Further inquiries can be directed to the corresponding authors.

## Ethics statement

The studies involving humans were approved by ethics committee UZA/UAntwerpen. The studies were conducted in accordance with the local legislation and institutional requirements. The participants provided their written informed consent to participate in this study.

## Author contributions

LD: Formal analysis, Investigation, Methodology, Writing – original draft. AT: Formal analysis, Investigation, Methodology, Writing – review & editing. EL: Conceptualization, Funding acquisition, Project administration, Writing – review & editing. AM: Writing – review & editing. KM: Conceptualization, Funding acquisition, Supervision, Writing – review & editing, Project administration.
